# Height of Nations: A Socioeconomic Analysis of Cohort Differences and Patterns among Women in 54 Low- to Middle-Income Countries

**DOI:** 10.1371/journal.pone.0018962

**Published:** 2011-04-20

**Authors:** S. V. Subramanian, Emre Özaltin, Jocelyn E. Finlay

**Affiliations:** 1 Department of Society, Human Development and Health, Harvard School of Public Health, Boston, Massachusetts, United States of America; 2 Department of Global Health and Population, Harvard School of Public Health, Boston, Massachusetts, United States of America; University of Illinois at Champaign-Urbana, United States of America

## Abstract

**Background:**

Adult height is a useful biological measure of long term population health and well being. We examined the cohort differences and socioeconomic patterning in adult height in low- to middle-income countries.

**Methods/Findings:**

We analyzed cross-sectional, representative samples of 364538 women aged 25-49 years drawn from 54 Demographic and Health Surveys (DHS) conducted between 1994 and 2008. Linear multilevel regression models included year of birth, household wealth, education, and area of residence, and accounted for clustering by primary sampling units and countries. Attained height was measured using an adjustable measuring board. A yearly change in birth cohorts starting with those born in 1945 was associated with a 0.0138 cm (95% CI 0.0107, 0.0169) increase in height. Increases in heights in more recent birth year cohorts were largely concentrated in women from the richer wealth quintiles. 35 of the 54 countries experienced a decline (14) or stagnation (21) in height. The decline in heights was largely concentrated among the poorest wealth quintiles. There was a strong positive association between height and household wealth; those in two richest quintiles of household wealth were 1.988 cm (95% CI 1.886, 2.090) and 1.018 cm (95% CI 0.916, 1.120) taller, compared to those in the poorest wealth quintile. The strength of the association between wealth and height was positive (0.05 to 1.16) in 96% (52/54) countries.

**Conclusions:**

Socioeconomic inequalities in height remain persistent. Height has stagnated or declined over the last decades in low- to middle-income countries, particularly in Africa, suggesting worsening nutritional and environmental circumstances during childhood.

## Introduction

Although height is highly heritable, improvements in attained height over time underscore the importance of environmental factors including nutrition, exposure to infections, and socioeconomic status, especially during childhood [1], [2], [3], [4], [5]. Consequently evaluating the changes in height over time across countries provides critical insights into the variations in childhood living conditions across countries. Further, socioeconomic inequalities in attained height within and across countries reveal the intergenerational nature of the distribution of conditions that influence health and well-being. Height has been shown to predict subsequent socioeconomic status [6], [7], morbidity [8], and mortality [9], [10]. Indeed, a mother's attained height has been shown to also be a strong risk factor of her offspring's mortality and growth failure extending into early childhood [11], [12]. Viewed this way, height is a stable and useful biological measure of standard of living [2], [13], that captures both current and future inequalities in population health. There are few cross-national assessments of adult height, with assessments largely confined to developed countries [14], [15], [16], [17]. Similar cross-national assessments of developing countries have been limited [18], and have not considered the socioeconomic inequalities in the patterning and changes in height over time. Using the largest available, nationally representative, and sample from 54 comparable surveys conducted in low- to middle-income countries with objective measurements of height, we provide an epidemiologic assessment of changes in height over a 40 year period along with its socioeconomic patterning both within and across countries.

## Methods

### Data Sources

Information from Demographic and Health Surveys (DHS) conducted in 54 countries between 1994 and 2008 provided the data for this study (**Table 1**) [19]. The DHS are nationally representative household sample surveys that measure population, health, socio-economic, and anthropometric indicators, emphasizing maternal and child health [20]. The DHS involves randomly selecting households within a cluster, and then within each household, women eligible for a more detailed individual survey are identified. Typically, these are women between the ages of 15–49. In a limited number of cases, women aged 10–49 are considered eligible, or in some earlier surveys the individual survey was limited to ever-married women. The DHS are important data source for studying population health across low- to middle-income countries due to extensive coverage, comparability, and data quality [21], [22], [23]. To ensure standardization and comparability across diverse sites and time, DHS surveys employ intense interviewer training, standardized measurement tools and techniques, an identical core questionnaire, and instrument pretesting [24]. Country reports detail pretesting and quality assurance measures by survey (see www.measuredhs.com/pubs/search/search_results.cfm?Type=5&srchTp=type&newSrch=1). The DHS is modular in structure, comprising a core questionnaire, a set of country-relevant sections, and country-specific variables. The DHS provides data with standardized variables across surveys and imputed dates of key events (see www.measuredhs.com/pubs/pdf/DHSG4/Recode4DHS.pdf). A multistage stratified cluster design with probabilistic sampling, with each unit of selection having a defined, and non-zero, probability of selection is employed for the DHS [25]. Every survey is stratified by urban and rural status and additionally by country-specific geographic or administrative regions. Detailed sampling plans are available from survey final reports at www.measuredhs.com/pubs/search/search_results.cfm?Type=5&srchTp=type&newSrch=1. **Table 1** describes each survey by country and year, along with sampling characteristics, response rates and sample sizes.

**Table 1:** Survey year, sample size, and mean height and year of birth for adult women.

Country	Survey Year	Response Rate	Sample Size	No. of PSUs	Mean Height	SD Height	Mean Birth Year	Percent Urban	Percent No Education
Total	1994–2008	92.7	364,538	31190	155.8	7.2	1970.2	45.2	33.4
Armenia	2005	92.8	4,218	308	158.1	5.7	1967.5	70.1	0.1
Azerbaijan	2006	95.5	5,412	318	158.4	5.9	1969.0	52.9	1.3
Bangladesh	2007	97.8	7,368	361	150.6	5.5	1971.5	39.0	40.7
Benin	2006	93.6	11,015	750	159.3	6.5	1972.0	40.3	73.3
Bolivia	2003	94.4	10,302	999	151.8	5.9	1972.4	62.4	6.7
Brazil	1996	75.1	2,264	777	155.7	6.6	1964.5	76.5	8.5
Burkina Faso	2003	95.7	7,337	400	161.6	6.2	1967.7	20.0	87.1
Cambodia	2005	91.2	5,081	557	152.4	5.4	1968.7	23.3	28.0
Cameroon	2004	92	2,816	463	160.4	6.3	1969.5	46.2	24.3
Central African Republic	1994	97.4	1,408	230	158.9	6.6	1962.8	37.1	55.3
Chad	2004	94.7	2,393	196	162.6	6.4	1971.8	41.6	76.7
Colombia	2004	81.7	22,947	3812	155.0	6.2	1968.6	76.4	4.3
Comoros	1996	95.4	644	99	154.8	5.8	1963.8	24.4	69.4
Congo, Dem. Rep.	2005	94	2,727	300	157.7	8.0	1972.3	45.6	24.4
Congo, Rep.	2007	96	3,922	225	159.0	8.1	1970.7	67.4	8.3
Cote d'Ivoire	1998	94.4	1,600	140	159.8	6.2	1964.4	63.9	56.1
Dominican Republic	1996	91.1	4,763	395	156.4	6.3	1960.8	59.9	12.3
Egypt, Arab Rep.	2008	98.8	13,183	1264	159.5	6.0	1972.0	42.9	36.1
Ethiopia	1997	94.2	3,868	534	157.6	6.6	1970.8	26.7	71.9
Gabon	2000	91.4	1,576	249	158.4	6.2	1967.9	60.1	6.3
Ghana	2008	95.1	2,958	411	159.3	6.7	1972.8	43.2	33.0
Guatemala	1998	78.1	1,773	276	147.3	6.3	1966.0	24.9	43.1
Guinea	2005	92.6	2,563	295	158.8	6.3	1969.8	24.3	87.2
Haiti	2005	98.4	2,932	339	158.6	6.5	1970.3	47.0	37.9
Honduras	2005	90.3	11,219	1046	152.0	6.4	1970.6	40.7	10.6
India	2005	92.3	74,291	3849	152.1	5.9	1970.9	45.5	39.7
Jordan	2007	96.7	4,484	464	158.2	6.6	1971.1	68.1	7.9
Kazakhstan	1999	95.9	1,600	205	159.8	6.3	1962.6	61.7	0.5
Kenya	2003	90.5	4,856	398	159.4	7.3	1973.8	30.6	17.6
Kyrgyz Republic	1997	96.7	2,424	162	158.0	5.8	1961.4	39.8	0.1
Lesotho	2004	89.8	1,879	404	157.6	6.7	1968.3	29.7	3.6
Liberia	2006	92.5	4,281	298	157.3	6.2	1971.8	41.2	53.9
Madagascar	2003	93.1	5,024	594	154.3	6.0	1973.6	25.9	23.2
Malawi	2004	93.6	6,182	521	156.2	6.3	1970.0	12.0	34.4
Mali	2006	95.4	8,676	407	161.4	6.7	1971.5	31.5	84.2
Moldova	2005	90.5	4,757	400	161.2	6.2	1967.5	56.9	0.3
Morocco	2003	95.1	10,334	480	158.5	6.0	1967.4	55.0	62.5
Mozambique	2003	86.2	6,912	604	156.0	6.2	1968.2	37.7	44.0
Namibia	2006	92.6	5,575	500	160.7	7.1	1971.7	46.5	10.9
Nepal	2006	98	6,280	260	150.8	5.5	1970.7	27.2	70.2
Nicaragua	2001	87.4	7,261	610	153.7	6.1	1965.7	57.1	22.3
Niger	2006	93.6	2,819	342	160.8	6.0	1971.8	31.7	82.5
Nigeria	2003	94.1	20,205	886	158.4	7.2	1973.6	31.3	45.1
Peru	2003	94.9	17,770	1293	151.3	5.7	1971.0	63.4	5.1
Rwanda	2005	97.9	3,202	462	157.7	6.5	1969.7	21.5	30.0
Senegal	2005	92.3	2,533	376	163.0	6.7	1970.4	41.1	70.0
Swaziland	2006	89.2	2,612	274	159.1	6.3	1971.0	33.8	12.0
Tanzania	2004	96.1	6,033	475	156.6	6.5	1969.8	23.3	27.6
Togo	1998	94.3	2,728	284	159.0	6.1	1965.9	21.4	65.7
Turkey	2003	88.8	2,393	645	156.4	5.6	1972.5	71.1	23.4
Uganda	2006	92.3	1,666	368	159.2	6.5	1971.5	14.5	29.2
Uzbekistan	1996	95.6	2,635	168	159.9	6.1	1960.9	54.1	0.1
Zambia	2007	94.4	4,091	319	158.5	6.5	1972.9	41.2	13.0
Zimbabwe	2005	85.6	4,746	398	160.3	6.2	1970.6	33.4	7.4

Note: PSU  =  Primary Sampling Units, SD  =  Standard Deviation.

### Study population and sample size

The study population comprises women (*n* = 454272) aged 25–49 years. There were 89577 women (19.72% of the sample) for whom height was intentionally not measured. Among those for whom height should have been measured, 4050 (<1%) did not have a height measure in the data, and a further 136 women (<1%) had implausible or extreme values (less than 100 cm or greater than 200 cm). One hundred and fifty seven observations (<1%) were missing data on covariates. The final analytical sample was 364538 women surveyed and measured between 1994 and 2008 in 54 countries.

### Outcome

Attained height (expressed in centimeters) was specified as a continuous outcome. Trained investigators measured each woman using an adjustable board calibrated in millimeters, and theoretically accurate to 1 millimeter [24].

### Independent Variables

Year of birth, household wealth, education, and place of residence (urban or rural) were the key independent variables (**Table 2**). Education was specified as having no schooling or incomplete primary, complete primary schooling, or having completed secondary or higher schooling. Household wealth was defined in terms of ownership of material possessions [26], with each woman assigned a wealth score based on a combination of different household characteristics that were weighted according to a factor analysis procedure. For this procedure, z-scores were calculated for each indicator variable and a principle components analysis was performed using these z-scores. For each household, the values of the indicator variables were multiplied by the factor loadings and summed to produce a standardized household index value with a mean of 0 and a standard deviation of 1. This standardized score was then divided into quintiles for each country [27], [28].

**Table 2:** Frequency and percentage distribution of sample by independent variables, and mean height by categories of independent variables for adult women.

	Frequency	Percent	Mean Height	SD
Total	364538	100%	155.8	7.2
**Wealth**				
Poorest Quintile	64387	17.7%	155.2	7.4
Second Poorest Quintile	67986	18.6%	155.2	7.2
Middle Quintile	72309	19.8%	155.6	7.1
Second Richest Quintile	75499	20.7%	156.0	7.1
Richest Quintile	84357	23.1%	157.0	7.1
**Schooling**				
None	121618	33.4%	155.8	7.6
Primary	100472	27.6%	154.9	7.1
Secondary or Higher	142448	39.1%	156.6	6.9
**Residence**				
Rural	199815	54.8%	155.6	7.3
Urban	164723	45.2%	156.2	7.1

Note: SD  =  Standard Deviation.

### Analysis

Individual country files were created ensuring consistency of variable definitions across countries. We used three types of analytical strategies. First, we conducted a pooled analysis of all the individual data from all countries with height modeled as a function of birth cohort (specified as year of birth), household wealth quintiles, highest educational attainment, urban/rural residence, and country fixed effects in a linear regression model, adjusted for clustering of the individual data by primary sampling units within countries with robust standard errors. The results from these models were used to draw global inference about the association between year of birth, household wealth, and height across all countries. We also specified and tested for interactions between household wealth quintiles and year of birth to assess whether the cohort differences in heights varied by household wealth. Second, we repeated the above strategy separately for every country to estimate the country-specific cohort differences, socioeconomic patterning in addition to assessing the interaction between wealth and year of birth in each country. Finally, since the pooled individual data yields a multilevel data structure of women at level-1 nested within primary sampling units (PSU) at level-2 nested within countries at level-3, we additionally estimated a multilevel linear regression to model the variation in height (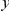) for a woman 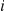 in PSU 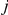 in country 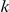 [29], as 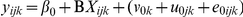, where 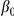 represented the mean height for the reference groups (*i.e.*, rural women born in 1945 with no schooling and in poorest wealth quintile) across all countries; and 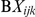 represents a vector of regression coefficients associated with variables year of birth, schooling categories, household wealth quintiles, and urban residence. The terms inside the brackets represents random effects associated with country 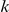 (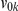), PSU 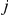 (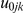), and a residual term for every individual 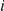 (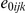). Assuming a normal distribution with a 0 mean, the model estimated a variance at level-1 (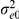: between-individual), level-2 (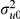: between-PSU), and level-3 (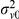: the between-country) in height. Results from the multilevel models were used to partition the variation in height attributable to individuals, primary sampling units (that are typically urban neighborhoods or rural villages), and countries [29]. Consequently, the two levels of environmental context in our study were countries, and PSUs within countries. The PSUs are typically smaller scales of geographically delineated administrative units, and as such represents within-country variation that is not attributable to individuals. Regression models were estimated using STATA ver.11.1MP (for the pooled and country specific models) [30], and MLwiN 2.20 (for multilevel models) [31].

### Ethical Review

The DHS data collection procedures were approved by the ORC Macro (Calverton, Maryland) Institutional Review Board as well as by the relevant body in each country which approves research studies on human subjects. Oral informed consent for the interview/survey was obtained from respondents by interviewers. The study was reviewed by Harvard School of Public Health Institutional Review Board and was considered exempt from full review because the study was based on an anonymous public use data set with no identifiable information on the survey participants.

## Results

**Figure 1:**
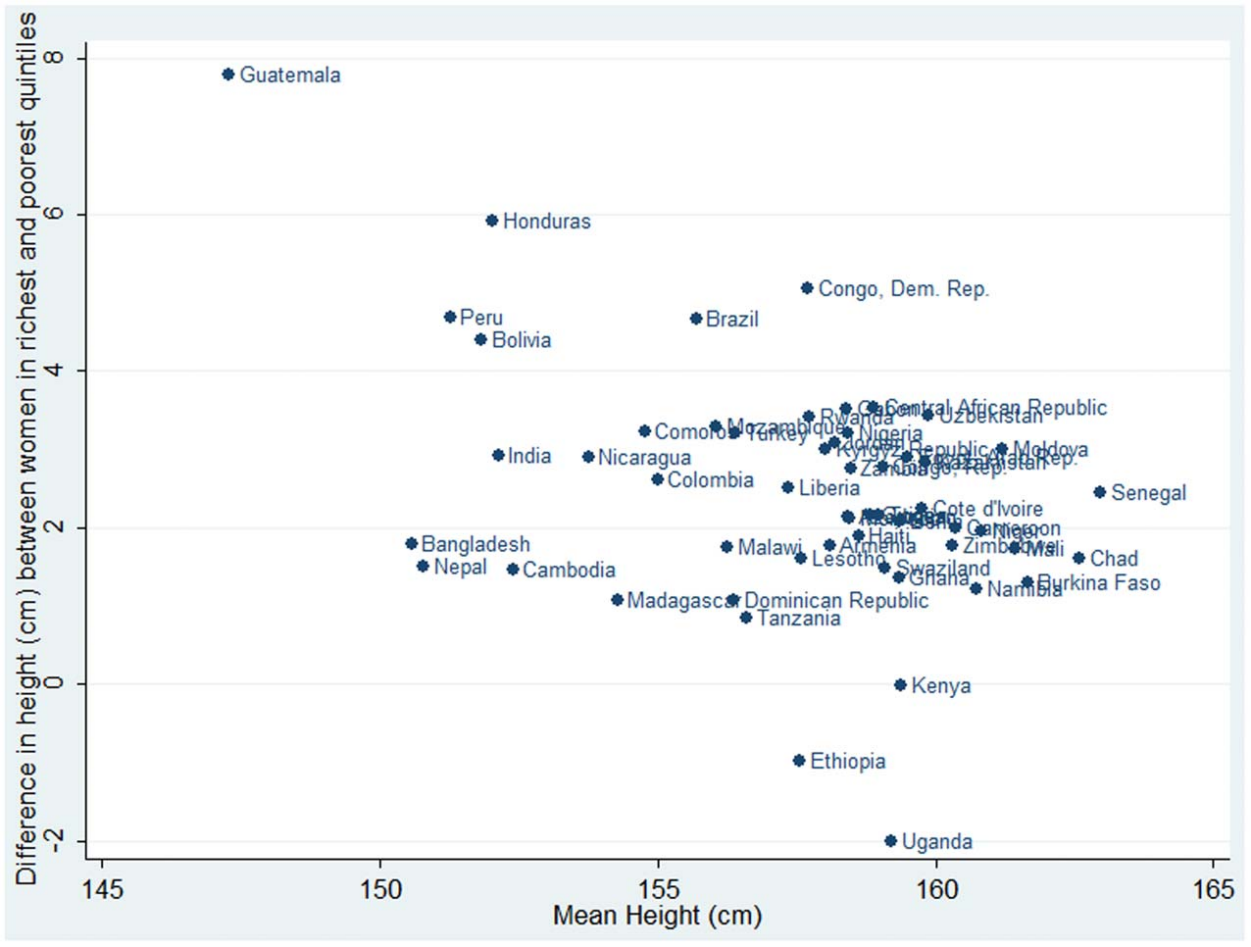
Country-level scatter plot between mean height (y axis) and gap in height between the richest and poorest wealth quintile (x axis) among adult women.

The pooled mean height in sample was 155.8 cm (SD 7.2), and mean height varied between 147.3 cm (SD 6.3) in Guatemala and 163.0 cm (SD 6.7) in Senegal (**Table 1**). 33.4% of the sample did not have any schooling, and 45.2% lived in urban areas (**Table 2**). In pooled samples, women in richer households, women who were more educated, or women who lived in urban areas were taller (**Table 2**). There was a strong negative country-level correlation between mean height and the differences in height between the richest and poorest wealth quintile (r  =  −0.4242, p = 0.0014) such that countries that are shorter on average also tend to have smaller gaps in height between the richest and the poorest wealth quintile (**Figure 1**).

In mutually adjusted pooled models, on average, height increased by 0.0138 cm (95% CI 0.0107, 0.0169) with one increase in year of birth (**Table 3**). There was a strong positive association between height and household wealth; those in top two quintile of household wealth were 1.988 cm (95% CI 1.886, 2.090) and 1.018 cm (95% CI 0.916, 1.120) taller, compared to those in the poorest wealth quintile. Women with primary schooling or secondary schooling were 0.159 cm (95% CI 0.0906, 0.227) and 1.328 cm (95% CI 1.252, 1.404) taller, respectively, compared to those with no schooling (**Table 3**). Urban-rural differentials in height were inconsequential in magnitude once the model was adjusted for women's year birth, education and wealth (**Table 3**).

**Table 3:** Unadjusted and mutually adjusted effects of year of birth, wealth quintiles, schooling, and place of residence on height accounting for within and between country variation for adult women.

	Unadjusted		Adjusted	
	Coefficient	95% CI	Coefficient	95% CI
Year of Birth	0.0249***	(0.020–0.027)	0.0138***	(0.009–0.016)
**Wealth**				
Poorest Quintile	(ref)	(ref)	(ref)	(ref)
Second Poorest Quintile	0.375***	(0.296–0.453)	0.287***	(0.208–0.365)
Middle Quintile	0.823***	(0.741–0.904)	0.609***	(0.524–0.693)
Second Richest Quintile	1.409***	(1.325–1.492)	1.018***	(0.927–1.108)
Richest Quintile	2.663***	(2.577–2.748)	1.988***	(1.886–2.089)
**Education**				
None	(ref)	(ref)	(ref)	(ref)
Primary	0.503***	(0.435–0.570)	0.159***	(0.090–0.227)
Secondary or Higher	2.286***	(2.215–2.356)	1.328***	(1.251–1.404)
**Residence**				
Rural	(ref)	(ref)	(ref)	(ref)
Urban	1.151***	(1.085–1.216)	−0.0836**	(−0.15–−0.00)

Note: Reference group are women born in 1945 or earlier who is in the lowest wealth quintile, with no education, and lives in a rural area.

*** p<0.01,

** p<0.05,

CI  =  Confidence Interval, ref  =  Reference.

**Figure 2:**
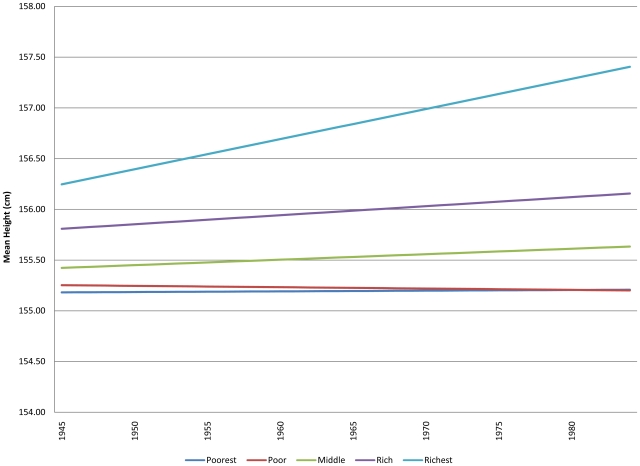
Predicted association between height and year of birth across wealth quintiles among adult women.

There was a substantial interaction effect between year of birth and wealth quintile (p = 0.0015), with the annual increase in height being largely restricted to the wealthiest quintile with stagnation in height for the poorest two wealth quintiles (**Figure 2**).

**Figure 3:**
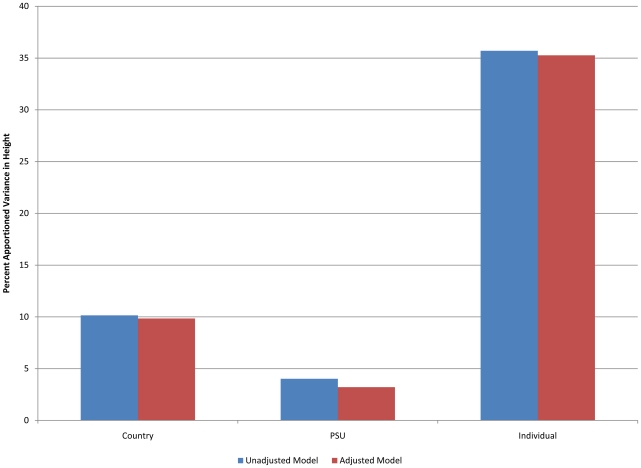
Percent of variation in height attributable to the individual and context (primary sampling units (PSU) and country) before and after accounting for the distribution of individual characteristics among adult women.

Of the total variation in height, 14.07% was attributable to environments (10.82% for countries, and 3.25% to primary sampling units) (**Figure 3**). The adjustment for year of birth, years of schooling, household wealth and urban/rural residence did not alter the apportioning of the total variation in height to different levels. Countries varied between a lower and upper bound of 149.3 cm and 162.2 cm respectively, while individuals varied between 144.2 cm and 167.3 cm around the mean of 155.75 cm (**Table 4**).

**Table 4:** Mean height and 95% Coverage Bounds in heights (in cm) between individuals, between-PSUs, and between-countries after accounting for within-country covariates.

Level	95% Lower Bound	Mean Height	95% Upper Bound
Country	149.30	155.75	162.20
PSU	152,22	155.75	159.28
Individual	144.20	155.75	167.30

Note: The height values were calculated by adding or subtracing 1.96 times the square root of the variance at each level to the global mean height (155.75 cm) from the adjusted model for the reference group. PSU  =  Primary Sampling Unit.

### Country-Specific Findings

Country-specific variability around the average was considerable; of the 54 countries, 26 countries were significantly taller than the global (pooled sample) average, and 14 were significantly (α = 0.05) shorter (**Table 5**). Senegal, Chad, Burkina Faso, Mali, and Niger were the five tallest countries while Guatemala, Bangladesh, Peru, Nepal, and Bolivia were the five countries where women exhibited the most substantially shorter height, compared to the global mean height (**Table 5**).

**Table 5:** Country specific differentials in height around the global mean for adult women.

	Unadjusted			Adjusted	
Country	Residual	SE	Country	Residual	SE
Guatemala	-10.0618	0.4900	Guatemala	-9.3937	0.4832
Bangladesh	-6.8609	0.4698	Bangladesh	-6.8546	0.4632
Nepal	-6.6276	0.4746	Peru	-6.5526	0.4564
Peru	-6.1421	0.4593	Nepal	-6.3379	0.4686
Bolivia	-5.6008	0.4626	Bolivia	-5.8320	0.4549
Honduras	-5.3563	0.4596	India	-5.5571	0.4642
India	-5.2983	0.4594	Honduras	-5.2022	0.4569
Cambodia	-5.0169	0.4672	Cambodia	-4.7504	0.4622
Nicaragua	-3.6937	0.4657	Nicaragua	-3.6147	0.4598
Madagascar	-3.1263	0.4674	Madagascar	-3.1686	0.4623
Comoros	-2.5932	0.5481	Colombia	-2.6809	0.4532
Colombia	-2.4361	0.4571	Comoros	-2.1004	0.5372
Brazil	-1.6297	0.4757	Brazil	-1.6689	0.4704
Mozambique	-1.4525	0.4653	Mozambique	-1.0571	0.4593
Malawi	-1.2054	0.4671	Dominican Republic	-0.9014	0.4664
Dominican Republic	-1.0993	0.4713	Turkey	-0.8753	0.4715
Tanzania	-0.9273	0.4677	Malawi	-0.8571	0.4626
Turkey	-0.9147	0.4762	Tanzania	-0.6835	0.4631
Liberia	-0.1140	0.4759	Kyrgyz Republic	-0.0365	0.4863
Ethiopia	0.1358	0.4700	Armenia	0.0001	0.4693
Lesotho	0.1617	0.4834	Lesotho	0.0307	0.4775
Congo (DRC)	0.1963	0.4802	Liberia	0.1938	0.4692
Rwanda	0.3162	0.4729	Congo (DRC)	0.1939	0.4734
Armenia	0.7087	0.4750	Azerbaijan	0.3645	0.4670
Jordan	0.7809	0.4698	Ethiopia	0.3918	0.4645
Kyrgyz Republic	0.8278	0.4943	Jordan	0.4069	0.4643
Zambia	0.9475	0.4754	Rwanda	0.5996	0.4680
Morocco	1.0257	0.4660	Zambia	0.9769	0.4679
Azerbaijan	1.0271	0.4730	Nigeria	1.1400	0.4561
Nigeria	1.0796	0.4578	Congo (Brazzaville)	1.1745	0.4731
Gabon	1.1772	0.4945	Gabon	1.2113	0.4876
Haiti	1.1951	0.4776	Swaziland	1.2333	0.4752
Guinea	1.4483	0.4814	Haiti	1.3240	0.4716
Central African Republic	1.5477	0.4981	Morocco	1.3293	0.4607
Togo	1.6562	0.4840	Kazakhstan	1.5613	0.4896
Swaziland	1.6690	0.4820	Uzbekistan	1.7057	0.4848
Congo (Brazzaville)	1.6740	0.4805	Ghana	1.7730	0.4689
Uganda	1.7455	0.4859	Kenya	1.8194	0.4651
Ghana	1.9017	0.4748	Egypt	1.9463	0.4567
Benin	1.9507	0.4629	Uganda	1.9522	0.4801
Kenya	1.9660	0.4710	Guinea	1.9882	0.4753
Egypt	2.1117	0.4599	Central African Republic	2.0505	0.4908
Cote d'Ivoire	2.4819	0.5060	Togo	2.1559	0.4773
Kazakhstan	2.4944	0.4968	Benin	2.3481	0.4562
Uzbekistan	2.6402	0.4925	Cote d'Ivoire	2.5316	0.4974
Zimbabwe	2.8824	0.4719	Zimbabwe	2.6190	0.4657
Cameroon	2.9191	0.4762	Cameroon	2.9256	0.4701
Namibia	3.2541	0.4680	Moldova	2.9266	0.4658
Niger	3.4021	0.4779	Namibia	2.9393	0.4626
Moldova	3.8618	0.4712	Niger	3.7006	0.4720
Mali	4.0485	0.4672	Mali	4.5063	0.4632
Burkina Faso	4.2414	0.4690	Burkina Faso	4.7695	0.4631
Chad	5.2039	0.4895	Chad	5.3883	0.4820
Senegal	5.4757	0.4789	Senegal	5.9500	0.4727

Note: Countries are presented from shortest to tallest differential from the global mean; Adjusted  =  adjusted for year of birth, household wealth, education, and place of residence (urban or rural); SE  =  Standard Error.

There was considerable variation in the association between year of birth and height across countries (**Table 6**). Of the 54 countries, 14 experienced a decrease in height in recent birth cohorts, with 7 countries experiencing a decline of over 0.05 cm decrease per year. Further, 39% (21/54) of the countries experienced no significant change, and 33% (19/54) experienced an increase in height ranging between 0.0203 (95% CI 0.0015, 0.0391) in Bangladesh and 0.0926 (95% CI 0.0446, 0.1410) in Kazakhstan. All 14 countries experiencing a decrease in height were in Africa (**Table 7**). Meanwhile, increases in height were observed in the Europe, Eastern Mediterranean, South-East Asia and Western Pacific regions, with the exception of Cambodia which experienced no significant change. About half (4/9) of the countries in South America had no significant change while the others experienced significant increases in height since 1945.

**Table 6:** Change in height for a 1-year increase in birth year and a 1 quintile increase in wealth from separate models for adult women.

Country	Year of Birth Slope	95% CI	Country	Wealth Slope	95% CI
Pooled	0.0130***	(0.009–0.016)	Pooled	0.464***	(0.439–0.488)
Rwanda	-0.104***	(-0.13–-0.07)	Uganda	-0.874***	(-1.14–-0.59)
Zambia	-0.0837***	(-0.11–-0.05)	Ethiopia	-0.480***	(-0.71–-0.24)
Comoros	-0.0760*	(-0.16–0.009)	Namibia	-0.0813	(-0.29–0.133)
Madagascar	-0.0752***	(-0.09–-0.05)	Kenya	-0.0169	(-0.24–0.225)
Congo (DRC)	-0.0535**	(-0.09–-0.00)	Madagascar	0.0500	(-0.11–0.217)
Mozambique	-0.0533***	(-0.07–-0.03)	Tanzania	0.0656	(-0.12–0.253)
Nigeria	-0.0589***	(-0.07–-0.04)	Burkina Faso	0.1020	(-0.03–0.240)
Chad	-0.0436**	(-0.08–-0.00)	Swaziland	0.1070	(-0.13–0.352)
Namibia	-0.0486***	(-0.07–-0.01)	Niger	0.1170	(-0.10–0.336)
Benin	-0.0368***	(-0.05–-0.01)	Malawi	0.121*	(-0.01–0.253)
Liberia	-0.0368***	(-0.06–-0.00)	Dominican Republic	0.1370	(-0.03–0.308)
Malawi	-0.0324***	(-0.05–-0.00)	Congo (Brazzaville)	0.1420	(-0.27–0.555)
Niger	-0.0328*	(-0.06–0.003)	Zimbabwe	0.1540	(-0.06–0.377)
Congo (Brazzaville)	-0.0186	(-0.05–0.019)	Togo	0.1810	(-0.04–0.406)
Mali	-0.0197*	(-0.03–0.000)	Bangladesh	0.192***	(0.068–0.315)
Uganda	-0.0103	(-0.05–0.036)	Cambodia	0.205***	(0.083–0.326)
Zimbabwe	-0.0150	(-0.04–0.013)	Mali	0.224***	(0.065–0.382)
Cambodia	0.0008	(-0.02–0.022)	Armenia	0.239**	(0.056–0.421)
Cameroon	-0.0024	(-0.03–0.033)	Ghana	0.259*	(-0.00–0.523)
Guatemala	-0.0096	(-0.05–0.04)	Nepal	0.280***	(0.146–0.413)
Haiti	-0.0039	(-0.03–0.031)	Morocco	0.280***	(0.115–0.444)
Honduras	-0.0051	(-0.02–0.011)	Colombia	0.305***	(0.211–0.398)
Nicaragua	0.0000	(-0.02–0.020)	Benin	0.331***	(0.198–0.463)
Swaziland	-0.0024	(-0.03–0.032)	Lesotho	0.345**	(0.072–0.617)
Togo	-0.0093	(-0.05–0.032)	Chad	0.355**	(0.037–0.672)
Ethiopia	0.0088	(-0.02–0.038)	Cote d'Ivoire	0.360*	(-0.04–0.765)
Burkina Faso	0.0112	(-0.01–0.032)	Rwanda	0.367***	(0.182–0.551)
Guinea	0.0148	(-0.01–0.045)	Nigeria	0.373***	(0.248–0.497)
Tanzania	0.0149	(-0.00–0.039)	Congo (DRC)	0.393**	(0.036–0.749)
Bangladesh	0.0203**	(0.001–0.039)	Zambia	0.397***	(0.157–0.636)
Lesotho	0.0230	(-0.01–0.064)	Haiti	0.407***	(0.130–0.683)
Brazil	0.0242	(-0.02–0.077)	Liberia	0.421***	(0.211–0.630)
Ghana	0.0249	(-0.00–0.058)	Azerbaijan	0.434***	(0.247–0.620)
India	0.0291***	(0.022–0.035)	CAR	0.516***	(0.163–0.868)
Gabon	0.0309	(-0.02–0.086)	Cameroon	0.542***	(0.281–0.802)
Nepal	0.0325***	(0.010–0.054)	Nicaragua	0.551***	(0.374–0.727)
Senegal	0.0356*	(-0.00–0.071)	Guinea	0.555***	(0.31–0.8)
Kyrgyz Republic	0.0367**	(0.004–0.069)	Turkey	0.559***	(0.367–0.750)
Cote d'Ivoire	0.0379	(-0.01–0.087)	Moldova	0.583***	(0.387–0.779)
Morocco	0.0371***	(0.021–0.052)	Kyrgyz Republic	0.591***	(0.365–0.816)
CAR	0.0397	(-0.03–0.113)	Jordan	0.620***	(0.458–0.781)
Uzbekistan	0.0404**	(0.006–0.074)	Mozambique	0.646***	(0.486–0.805)
Peru	0.0418***	(0.029–0.053)	Gabon	0.663***	(0.339–0.986)
Dominican Republic	0.0444***	(0.018–0.070)	Egypt	0.679***	(0.555–0.802)
Bolivia	0.0483***	(0.031–0.064)	Comoros	0.718***	(0.376–1.059)
Kenya	0.0489***	(0.017–0.080)	Senegal	0.728***	(0.437–1.018)
Egypt	0.0523***	(0.036–0.067)	Bolivia	0.764***	(0.616–0.911)
Azerbaijan	0.0531***	(0.029–0.076)	Uzbekistan	0.765***	(0.500–1.029)
Armenia	0.0591***	(0.035–0.082)	India	0.839***	(0.782–0.895)
Colombia	0.0658***	(0.054–0.077)	Guatemala	0.863***	(0.494–1.231)
Turkey	0.0825***	(0.034–0.130)	Kazakhstan	0.881***	(0.526–1.235)
Jordan	0.0837***	(0.052–0.114)	Brazil	0.900***	(0.660–1.139)
Moldova	0.0869***	(0.063–0.110)	Peru	0.912***	(0.800–1.023)
Kazakhstan	0.0926***	(0.044–0.140)	Honduras	1.132***	(0.983–1.280)

Note:

*** p<0.01,

** p<0.05,

* p<0.1; CI = Confidence Interval.

**Table 7:** Summary table showing frequency and percentage of countries across different WHO regions that experienced a statistically significant decline, or no change or increase in height among adult women.

		Decline*		No Change*		Increase*	
WHO Region**	N	N	Percent	N	Percent	N	Percent
Total	54	14	25.9	21	38.9	19	33.3
Africa	31	14	45.2	15	48.4	2	6.5
Americas	9	0	0.0	5	55.6	4	44.4
Eastern Mediterranean	3	0	0.0	0	0.0	3	100.0
Europe	7	0	0.0	0	0.0	7	100.0
South-East Asia/West Pacific	4	0	0.0	1	25.0	3	75.0

Note:

* Based on significance at p = 0.05

** **Africa**: Benin, Burkina Faso, Cameroon, Central African Republic, Chad, Comoros, Congo (Brazzaville), Congo (DRC), Cote d'Ivoire, Ethiopia, Gabon, Ghana, Guinea, Kenya, Lesotho, Liberia, Madagascar, Malawi, Mali, Mozambique, Namibia, Niger, Nigeria, Rwanda, Senegal, Swaziland, Tanzania, Togo, Uganda, Zambia, Zimbabwe; **Americas**: Bolivia, Brazil, Colombia, Dominican Republic, Guatemala, Haiti, Honduras, Nicaragua, Peru; **Eastern Mediterranean**: Egypt, Jordan, Morocco; **Europe**: Armenia, Azerbaijan, Kazakhstan, Kyrgyz Republic, Moldova, Turkey, Uzbekistan; **South-East Asia/West Pacific**: Bangladesh, Cambodia, India, Nepal.

In 51 of 54 countries, there was a statistically significant (p<0.05) difference between the poorest two and richest two wealth quintiles in the association between year of birth and height (**Table 8**). In general, countries that experienced average decline in height across birth cohorts, the decline was substantially greater among the poorest two wealth quintiles. Conversely, countries that experienced a positive average increase in height, such increases were largely concentrated among the wealthier quintiles. For instance, in Brazil the heights of the wealthier two quintiles increased by 0.16 cm while those for the poorest two quintiles increased by 0.01 cm. In Mozambique, which experienced an overall decline in height, the annual decrease was −0.08 cm for the poorest two quintiles, and −0.02 cm for the richest two quintiles.

**Table 8:** Country-specific predicted effects of change in height for a 1-year increase in birth year in the two poorest and two richest wealth quintiles among adult women.

	Poorest/Second Poorest Quintile		Richest/Second Richest Quintile		
Country	Year of Birth Coefficient	95% CI	Year of Birth Coefficient	95% CI	p- value**
Pooled	-0.00035	(0–0.005)	0.0194***	(0.013–0.024)	0.000
Armenia	0.0427**	(0.001–0.084)	0.0749***	(0.037–0.11)	0.000
Azerbaijan	0.0549***	(0.02–0.088)	0.0681***	(0.03–0.105)	0.000
Bangladesh	0.0316*	(0–0.064)	0.0397***	(0.014–0.063)	0.000
Benin	-0.0486***	(-0.07–-0.01)	-0.0269*	(-0.04–0.007)	0.000
Bolivia	0.0621***	(0.036–0.087)	0.103***	(0.077–0.128)	0.000
Brazil	0.0196	(-0.04–0.085)	0.165***	(0.061–0.268)	0.000
Burkina Faso	-0.000864	(-0.03–0.035)	0.0431***	(0.011–0.074)	0.000
Cambodia	0.0165	(-0.01–0.05)	0.0127	(-0.01–0.041)	0.000
Cameroon	0.014	(-0.04–0.071)	-0.0118	(-0.06–0.043)	0.000
Central African Republic	0.0737	(-0.02–0.172)	0.0454	(-0.09–0.188)	0.000
Chad	-0.0736*	(-0.14–0.002)	-0.0211	(-0.07–0.031)	0.000
Colombia	0.0703***	(0.051–0.089)	0.115***	(0.097–0.132)	0.000
Comoros	-0.0582	(-0.16–0.049)	0.0084	(-0.13–0.146)	0.000
Congo (DRC)	-0.0243	(-0.09–0.048)	-0.0143	(-0.07–0.056)	0.000
Congo (Brazzaville)	0.0363	(-0.03–0.104)	-0.0267	(-0.07–0.031)	0.000
Cote d'Ivoire	0.046	(-0.04–0.137)	0.0377	(-0.02–0.101)	0.000
Dominican Republic	0.0760***	(0.04–0.111)	0.0582***	(0.014–0.101)	0.000
Egypt	0.0674***	(0.045–0.089)	0.0437***	(0.02–0.065)	0.000
Ethiopia	-0.0101	(-0.05–0.039)	0.034	(0–0.075)	0.540
Gabon	0.0347	(-0.03–0.108)	0.0772	(-0.03–0.186)	0.000
Ghana	0.0251	(-0.02–0.076)	0.0172	(-0.02–0.063)	0.000
Guatemala	0.0266	(-0.03–0.085)	0.0997	(-0.03–0.232)	0.000
Guinea	-0.00909	(-0.05–0.04)	0.033	(-0.01–0.084)	0.000
Haiti	0.0319	(-0.01–0.082)	0.0404	(-0.01–0.092)	0.000
Honduras	0.00563	(-0.01–0.03)	0.00893	(-0.01–0.032)	0.000
India	0.0160**	(0.003–0.028)	0.0427***	(0.033–0.05)	0.000
Jordan	0.0621***	(0.021–0.102)	0.113***	(0.059–0.166)	0.000
Kazakhstan	0.0598	(-0.02–0.145)	0.140***	(0.073–0.206)	0.000
Kenya	0.0723**	(0.014–0.13)	0.0264	(-0.01–0.07)	0.810
Kyrgyz Republic	0.0680**	(0.01–0.125)	9.64E-05	(-0.04–0.044)	0.000
Lesotho	0.016	(-0.05–0.083)	0.0251	(-0.03–0.083)	0.010
Liberia	-0.0336*	(-0.06–0.002)	-0.032	(-0.08–0.022)	0.000
Madagascar	-0.0751***	(-0.11–-0.03)	-0.0673***	(-0.09–-0.02)	0.000
Malawi	-0.0195	(-0.05–0.016)	-0.00973	(-0.03–0.035)	0.000
Mali	-0.0236	(-0.05–0.006)	-0.0273	(-0.05–0.013)	0.000
Moldova	0.0677***	(0.023–0.111)	0.107***	(0.074–0.139)	0.000
Morocco	0.0534***	(0.032–0.074)	0.0618***	(0.036–0.085)	0.000
Mozambique	-0.0834***	(-0.11–-0.04)	-0.0218	(-0.05–0.012)	0.000
Namibia	-0.0208	(-0.06–0.023)	-0.0202	(-0.06–0.024)	0.040
Nepal	0.0563***	(0.023–0.088)	0.0332**	(0.003–0.062)	0.000
Nicaragua	-0.00622	(-0.03–0.023)	0.0281*	(0–0.059)	0.000
Niger	-0.0258	(-0.07–0.029)	-0.0242	(-0.07–0.032)	0.000
Nigeria	-0.0499***	(-0.07–-0.02)	-0.0460***	(-0.06–-0.01)	0.000
Peru	0.0418***	(0.022–0.061)	0.0799***	(0.06–0.097)	0.000
Rwanda	-0.0984***	(-0.14–-0.05)	-0.0494*	(-0.09–0.012)	0.000
Senegal	0.0351	(-0.02–0.097)	0.0345	(-0.02–0.092)	0.000
Swaziland	-0.0267	(-0.08–0.032)	0.0441	(0–0.097)	0.030
Tanzania	0.0267	(-0.01–0.067)	0.0227	(-0.01–0.058)	0.090
Togo	0.00377	(-0.05–0.064)	-0.0462	(-0.11–0.038)	0.000
Turkey	0.0526	(-0.01–0.116)	0.0782*	(0–0.161)	0.000
Uganda	-0.0246	(-0.09–0.047)	0.0167	(-0.05–0.087)	0.000
Uzbekistan	0.0495*	(0–0.106)	0.0219	(-0.02–0.065)	0.000
Zambia	-0.0944***	(-0.14–-0.04)	-0.0517**	(-0.09–0)	0.000
Zimbabwe	0.0399*	(0–0.081)	0.0178	(-0.02–0.057)	0.000

Note: * t-test (Poorest/Second Poorest Quintile)  =  (Richest/Second Richest Quintile);

*** p<0.01,

** p<0.05,

* p<0.1: for the test that the coefficient is significantly different from zero.

The strength of the positive association between height and wealth also varied across countries; in 41 out of 54 countries there was a positive and statistically significant (á = 0.05) association for the effect of a change in wealth quartile on height, varying between 0.121 cm in Malawi to 1.132 cm in Honduras. The association between wealth and adult height, while positive, was not statistically significant in 9 countries. In Ethiopia and Uganda there was a statistically significant negative association between height and wealth such that women in wealthier households were shorter (**Table 6**).

## Discussion

The study has three salient findings. First, the birth cohort differences in attained heights among women living in low- to middle-income countries suggest a decline or stagnation in height in a majority of the countries. While decline in height was particularly concentrated in Africa, about half of the countries surveyed in South America showed stagnation. Second, cohort differences in height varied substantially by individuals' socioeconomic status both in pooled analysis as well as country-specific analysis, such that decline and stagnation are largely observed for socioeconomically disadvantaged groups, while the increases are concentrated among the socioeconomically advantaged groups. Finally, the positive association between socioeconomic status and attained height appears to be a consistent and universal pattern in low- to middle-income countries, with some heterogeneity in the strength of such an association.

Before we interpret these findings, we outline the limitations of our data. First, our assessment of increases or decreases in attained height in birth cohort differences is based upon self-reports of current age. Age reporting in low- to middle-income countries has been raised as an important concern because reporting of ages in multiples of five or ten is relatively common and leads to age clustering. However, an analysis of the quality of age reporting in the DHS for all surveys between 1985–2003 found that the overall quality of age-reporting was found to be high with most surveys exhibiting few problems [32]. The DHS also employs extensive imputation procedures and consistency checks to ensure that birth years and ages are as accurate as possible [33]. Our modeling of year of birth as a continuous variable, we believe, attenuates some of these concerns since we are not making inferences to birth cohorts born during specific periods, which will be more sensitive to misclassification of reported age. Second, the typical method of assessing changes in height using birth cohorts relies on the assumption that heights are not changing by age. We ensured this by considering only women between 25 and 49 years of age where we do not expect heights to change. Even though prior research suggests that girls attain height by age 20, we chose 25 as the lower conservative cut-point for age since the empirical data suggested that heights among 15–24 year olds was shorter than the other age groups suggesting that potentially height was yet to be attained (see **Figure S1**, and **Table S1**). At the other extreme it is known that humans also shrink with age [34], even though it is unlikely to significantly influence the age range considered in this study. Third, our assessment of women's socioeconomic status, through household wealth and education was measured at the same time when height was measured. We therefore make the assumption that the level of socioeconomic status (as captured through contemporary household wealth or education) is reflective of the socioeconomic status during the women's childhood and growing years. In addition, wealth can be a consequence of height through a variety of mechanisms [35]. Therefore, no causal interpretation of the effects associated with socioeconomic markers should be made based on this study. These important limitations are however offset by having measured height on representative samples of women from a large cross-section of low- to middle-income countries allowing a rich description of the global and country-specific changes and patterning of adult height, thus allowing insights into the level of inequalities in population health across and within countries.

Our findings related specifically to estimating overall birth cohort differences in height for countries are in agreement with an ecological study that examined changes in heights over time in low- to middle-income countries using the same data [18], and reported that while heights in Africa have been declining, they have been increasing in the rest of the developing world. Our study differed on one important methodological aspect. While we use the disaggregated individual data to estimate changes in height using year of birth as an explanatory variable in a regression model, the comparison study was ecological with average heights being computed for each birth year-country combination, and using this as the outcome and countries as the explanatory variable, weighted by the size of each group. Our study with the use of individual data overcomes the cross-level bias that characterizes aggregate analysis [36], [37]. Importantly, as our findings reveal, average descriptions of changes in height seem to also mask the important social inequalities in how heights have changed over the last forty years.

Twenty six percent (14/53) of the low- and middle-income countries included in this study experienced a significant (p = 0. 05) decline in attained height since 1945 while another 43% experienced no change in height over birth cohorts. This is driven entirely by declining heights in Africa; with half of countries in South America showing stagnation in height since 1945 and heights modestly increasing in the rest of the world. Importantly, in most cases, the increases within countries were restricted to the socioeconomically advantaged groups. This differs from evidence on changes in height in developed countries. For instance, the evidence from European countries showed a mostly positive increase in height over the last century, including during the same time period as this study [14], [15], [17], [38], [39], [40]. Likewise, even though there was stagnation in height growth between the years 1965–1974 for white adults in the United States, in subsequent years a positive trend in height has been reported [41]. At the same time, however, black women in the US experienced a decline in height [41]. Since black women in the US, on average, are of lower socioeconomic status *within* the US, it suggests a pattern similar to what we observe for lower socioeconomic groups within and among low- to middle-income countries. Other country-specific studies on height trends from developing countries from Turkey [42], Iran [43], India [44], and the United Arab Emirates [45], have reported a positive trend or increase in stature over time. Our results are consistent with the study from Turkey, and India, which are the two countries that overlap with our cross-national sample.

The association between socioeconomic status and height has consistently been shown across developed countries [14], [38], [39], [46]. A review on 10 European countries showed significant persistent education-related differences in height for both men and women: the range of differences for higher educated men was 1.6–3.0 cm, and women was 1.2–2.2 cm [14]. The results from our study indicated that a one quintile increase in wealth for 41 out of 54 countries was associated with an increase in height ranging from 0.3–1.5cm depending on the country. Focusing on the importance of the childhood environment to attained height, it seems that childhood conditions have not improved concurrent with improvements in infant mortality for those countries experiencing relatively few changes in height and may have actually worsened (or inequalities increased) for those exhibiting negative cohort differences given the results of year of birth estimates. Similarly, childhood conditions may be more unequal for countries that exhibited a steeper gradient in the association between socioeconomic status and height. At the same time, in 2 countries (Uganda and Ethiopia) the correlation between height and wealth was negative, which is contrary to the near-universal pattern of a positive association. Whether this finding represents a genuine exception or if it is artifact of a systematically biased measurement of height or wealth index remains a subject for further investigation.

In summary, a salient finding of our study is the decline or stagnation in attained heights over the last 40 years, particularly in Africa and for the poorer populations across the world. Increases in height are largely restricted to non-African better off populations. Thus, even though infant mortality and other acute childhood morbidities have decreased substantially over time in these countries, the stagnation and decline across cohorts in attained height suggest little improvements, and perhaps deterioration, in early childhood living conditions including nutritional and environmental circumstances. While our study focuses on attained height for women from birth years that preceded the concerted global efforts on improved childhood health and nutrition spurred by the Millennium Development Goals, it will be important to monitor the height patterns for subsequent birth cohorts in these populations. The persistent country differences in adult height even among recent birth cohorts indicate the intergenerational continuity in differences in childhood living conditions. The marked socioeconomic differentials observed consistently within every country suggest that health inequalities in these countries will be persistent in future. Further research is needed to understand the environmental determinants that enable realization of the potential height of a child, and especially a girl child, which in turn have substantial consequences for their own health and social well being as well as the health of their offspring.

## Supporting Information

Figure S1 Mean Height (cm) By Age Pooled Across Countries.
(DOCX)

Table S1 Mean Height (cm) By Age Groups, By Country.
(DOCX)
