# Supplementary material for: Height of Nations: A Socioeconomic Analysis of Cohort Differences and Patterns among Women in 54 Low- to Middle-Income Countries
Source: PLoS One. 2011 Apr 20;6(4):e18962. doi: 10.1371/journal.pone.0018962 (PMC3080396; doi:10.1371/journal.pone.0018962)
Supplement: Figure S1 — Mean Height (cm) By Age Pooled Across Countries. (DOCX) [file pone.0018962.s001.docx]

**Supplementary Figure 1: Mean Height (cm) By Age Pooled Across Countries**
