# Supplementary material for: Height of Nations: A Socioeconomic Analysis of Cohort Differences and Patterns among Women in 54 Low- to Middle-Income Countries
Source: PLoS One. 2011 Apr 20;6(4):e18962. doi: 10.1371/journal.pone.0018962 (PMC3080396; doi:10.1371/journal.pone.0018962)
Supplement: Table S1 — Mean Height (cm) By Age Groups, By Country. (DOCX) [file pone.0018962.s002.docx]

**Supplementary Table 1: Mean Height (cm) By Age Groups, By Country**

| Age Group | 15-19 | 20-24 | 25-29 | 30-34 | 35-39 | 40-44 | 45-49 |
| --- | --- | --- | --- | --- | --- | --- | --- |
|  |  |  |  |  |  |  |  |
| Armenia | 158.34 | 158.86 | 158.64 | 158.38 | 158.36 | 157.71 | 157.56 |
| Azerbaijan | 157.95 | 158.85 | 158.78 | 158.75 | 158.49 | 158.09 | 157.99 |
| Bangladesh | 150.45 | 150.60 | 150.82 | 150.72 | 150.56 | 150.49 | 149.92 |
| Benin | 156.61 | 157.93 | 158.81 | 159.55 | 159.78 | 159.71 | 159.27 |
| Bolivia | 153.45 | 153.10 | 152.81 | 151.85 | 151.54 | 151.30 | 151.04 |
| Brazil | 155.78 | 156.25 | 155.80 | 156.08 | 154.98 | 155.04 | 155.50 |
| Burkina Faso | 159.90 | 161.68 | 161.80 | 161.74 | 161.81 | 161.48 | 161.18 |
| Cambodia | 151.21 | 152.32 | 152.16 | 152.52 | 153.04 | 152.05 | 152.05 |
| Cameroon | 158.77 | 159.67 | 160.26 | 160.50 | 160.59 | 160.31 | 160.08 |
| Central African Republic | 157.93 | 158.68 | 158.92 | 159.67 | 158.11 | 157.14 | 158.91 |
| Chad | 161.24 | 161.65 | 162.52 | 162.29 | 162.99 | 162.73 | 162.61 |
| Colombia | 156.01 | 156.02 | 155.73 | 155.26 | 155.12 | 154.58 | 153.92 |
| Comoros | 152.86 | 154.74 | 154.61 | 154.25 | 155.79 | 154.87 | 153.02 |
| Congo, Dem. Rep. | 154.73 | 155.88 | 157.28 | 157.58 | 158.78 | 157.44 | 157.55 |
| Congo, Rep. | 157.14 | 158.35 | 158.87 | 159.10 | 159.50 | 159.08 | 158.50 |
| Cote d'Ivoire | 158.70 | 160.05 | 159.94 | 160.06 | 159.72 | 159.53 | 158.77 |
| Dominican Republic | 157.33 | 157.10 | 156.64 | 156.83 | 156.30 | 156.12 | 155.31 |
| Egypt, Arab Rep. | 158.58 | 159.39 | 159.85 | 159.80 | 159.57 | 159.20 | 158.79 |
| Ethiopia | 155.89 | 157.41 | 157.61 | 157.73 | 157.36 | 157.77 | 157.19 |
| Gabon | 157.39 | 158.46 | 158.65 | 158.31 | 158.47 | 157.49 | 158.61 |
| Ghana | 157.53 | 159.04 | 159.52 | 159.01 | 159.60 | 159.83 | 158.50 |
| Guatemala | 147.49 | 148.11 | 147.86 | 147.44 | 146.72 | 146.10 | 146.95 |
| Guinea | 157.87 | 158.75 | 158.85 | 159.13 | 158.90 | 158.66 | 158.37 |
| Haiti | 158.15 | 159.07 | 159.04 | 158.52 | 158.57 | 158.40 | 158.20 |
| Honduras | 152.78 | 152.68 | 152.30 | 151.96 | 151.91 | 151.99 | 151.80 |
| India | 152.05 | 152.35 | 152.28 | 152.30 | 152.10 | 152.07 | 151.65 |
| Jordan | 158.53 | 158.78 | 158.59 | 158.58 | 158.32 | 157.67 | 157.26 |
| Kazakhstan | 159.48 | 160.50 | 160.27 | 160.87 | 159.77 | 158.73 | 159.18 |
| Kenya | 157.61 | 159.04 | 159.30 | 160.08 | 159.20 | 159.56 | 158.28 |
| Kyrgyz Republic | 157.91 | 158.93 | 158.42 | 157.94 | 157.89 | 157.81 | 157.87 |
| Lesotho | 155.71 | 157.02 | 157.66 | 158.04 | 157.68 | 157.22 | 157.07 |
| Liberia | 155.29 | 156.37 | 156.90 | 157.46 | 157.77 | 157.49 | 157.23 |
| Madagascar | 151.64 | 153.04 | 153.54 | 153.96 | 154.58 | 154.86 | 155.09 |
| Malawi | 154.23 | 155.68 | 156.09 | 156.32 | 156.15 | 156.75 | 156.05 |
| Mali | 159.43 | 160.87 | 161.29 | 161.46 | 161.58 | 161.57 | 161.34 |
| Moldova | 161.80 | 162.43 | 161.93 | 162.02 | 161.07 | 161.10 | 160.12 |
| Morocco | 158.04 | 158.84 | 158.64 | 158.96 | 158.52 | 158.33 | 157.55 |
| Mozambique | 154.74 | 155.72 | 155.64 | 155.89 | 156.27 | 156.57 | 156.22 |
| Namibia | 159.37 | 160.15 | 160.35 | 161.07 | 160.59 | 161.13 | 160.53 |
| Nepal | 151.09 | 151.29 | 151.05 | 151.02 | 150.79 | 150.73 | 149.96 |
| Nicaragua | 153.75 | 154.31 | 153.86 | 153.74 | 153.66 | 153.92 | 153.47 |
| Niger | 158.26 | 160.65 | 160.70 | 160.54 | 161.19 | 160.77 | 161.07 |
| Nigeria | 155.30 | 157.27 | 158.00 | 158.49 | 158.69 | 158.93 | 158.41 |
| Peru | 152.04 | 152.21 | 151.73 | 151.67 | 151.21 | 151.05 | 150.32 |
| Rwanda | 154.10 | 156.50 | 157.02 | 157.63 | 157.81 | 158.54 | 158.12 |
| Senegal | 161.90 | 162.98 | 163.31 | 162.99 | 163.17 | 162.47 | 162.41 |
| Swaziland | 157.96 | 158.48 | 158.88 | 159.62 | 159.37 | 158.75 | 158.60 |
| Tanzania | 154.50 | 155.84 | 156.59 | 156.82 | 156.98 | 156.35 | 155.83 |
| Togo | 157.21 | 158.73 | 158.87 | 159.24 | 159.00 | 158.79 | 158.14 |
| Turkey | 158.24 | 157.07 | 156.88 | 156.42 | 155.71 | 155.52 | 151.91 |
| Uganda | 156.82 | 158.33 | 158.88 | 159.36 | 159.55 | 159.30 | 158.94 |
| Uzbekistan | 158.57 | 160.10 | 160.29 | 159.70 | 159.64 | 159.49 | 160.27 |
| Zambia | 155.67 | 157.33 | 157.91 | 158.49 | 158.68 | 159.10 | 158.97 |
| Zimbabwe | 159.04 | 159.77 | 160.44 | 160.43 | 160.24 | 160.36 | 159.85 |
